# Social deprivation, race, and metabolic syndrome in patients with polycystic ovary syndrome

**DOI:** 10.1210/jendso/bvag063

**Published:** 2026-03-20

**Authors:** Iris T Lee, Shakira King, Naria Sealy, John Rees, Sunni L Mumford, Stefanie N Hinkle, Anuja Dokras

**Affiliations:** Department of Obstetrics and Gynecology, University of Pennsylvania, Philadelphia, PA 19104, USA; Department of Obstetrics and Gynecology, University of Pennsylvania, Philadelphia, PA 19104, USA; Department of Biostatistics, Epidemiology & Informatics, University of Pennsylvania, Philadelphia, PA 19104, USA; Department of Obstetrics and Gynecology, University of Pennsylvania, Philadelphia, PA 19104, USA; Department of Obstetrics and Gynecology, University of Pennsylvania, Philadelphia, PA 19104, USA; Department of Biostatistics, Epidemiology & Informatics, University of Pennsylvania, Philadelphia, PA 19104, USA; Department of Biostatistics, Epidemiology & Informatics, University of Pennsylvania, Philadelphia, PA 19104, USA; Department of Obstetrics and Gynecology, University of Pennsylvania, Philadelphia, PA 19104, USA

**Keywords:** metabolic syndrome, polycystic ovary syndrome, Social Deprivation Index, social determinants of health

## Abstract

**Context:**

Social determinants of health (SDoH) are a key contributor to cardiovascular disease (CVD) risk, including metabolic syndrome (MetSyn), as well as racial disparities in that risk. It is unknown if SDoH are associated with MetSyn in women with polycystic ovary syndrome (PCOS), who have a high risk of CVD. Furthermore, it is unclear if SDoH contributes to the Black-White disparity in MetSyn among women with PCOS.

**Objective:**

To assess the association between the Social Deprivation Index (SDI; a proxy for SDoH) and the development of new-onset MetSyn in women with PCOS and whether SDI plays a role in the racial disparity in MetSyn.

**Design:**

Longitudinal cohort study.

**Setting:**

Tertiary care center.

**Patients or Other Participants:**

Women with hyperandrogenic PCOS and 2+ assessments for MetSyn 3 years apart.

**Intervention(s):**

None.

**Main Outcome Measure(s):**

Development of new-onset MetSyn; proportion of association between race and MetSyn attributable to SDI.

**Results:**

Two hundred twenty-two participants were followed for a median of 7 years; 43.7% developed new-onset MetSyn. High SDI, indicating greater social deprivation, was associated with an increased risk of developing MetSyn (adjusted relative risk 1.42, 95% confidence interval 1.07-1.91 adjusting for age), as was Black race. The proportion of the association between race and new-onset MetSyn explained by SDI was 21%.

**Conclusion:**

High social deprivation is associated with increased risk of new-onset MetSyn and may contribute to the higher risk in Black compared to White women with PCOS. These results highlight the importance of considering SDoH, particularly in an already high-risk population.

Polycystic ovary syndrome (PCOS) is a common endocrine disorder affecting up to 13% of reproductive-age women [1]. In addition to reproductive and psychological symptoms, it is well established that PCOS is associated with increased cardiometabolic risk, including obesity, hypertension, dyslipidemia, and type 2 diabetes. It is also associated with a 3-fold increased odds of metabolic syndrome (MetSyn), a constellation of cardiovascular disease (CVD) risk factors that predicts future myocardial infarction, stroke, and all-cause mortality [2]. Some studies report that the prevalence of MetSyn is as high as 43% in women with PCOS, nearly double the prevalence in the general US population [3]. Prior studies by the authors have found that depression, high androgens, and elevated body mass index (BMI) are associated with increased risk of MetSyn in women with PCOS [4, 5].

In the general population, social determinants of health (SDoH)—the nonmedical factors that influence health outcomes by affecting access to resources and limiting the ability to make healthy lifestyle choices—have emerged as a key contributor to CVD risk. For example, proximity to grocery stores is associated with a 17% lower prevalence of obesity, and lack of social support increases the risk of cardiac events by 2.5 times [6, 7]. Social deprivation, a measure of disadvantaged SDoH, has been found to be associated with increased CVD mortality as well as the prevalence of MetSyn [8, 9]. Mechanisms by which SDoH and social deprivation may impact MetSyn and ultimately CVD include chronic psychological and physical stress, physical inactivity, specific behaviors such as smoking, unhealthy dietary habits, and longstanding exposure to racism [10].

The role of SDoH and social deprivation in MetSyn among women with PCOS—a group at intrinsically high risk of CVD due to underlying pathophysiology—has been less well studied. A 2021 study by Berni et al examined women with PCOS in England using the Clinical Practice Research Datalink Aurum database and found that social deprivation increased the risk of progression to the composite endpoint of major adverse cardiovascular events [11]. Similar studies among women with PCOS have not been conducted in the United States, where there is a greater association between SDoH and CVD in the general population and more pronounced disparities in health outcomes by socioeconomic status [12]. Conventional approaches to decrease CVD in women with PCOS have focused on targeting the traditional risk factors, such as weight loss or improving insulin resistance. It is possible that there is heterogeneity in the effect of SDoH and social deprivation on MetSyn depending on baseline risk, and efforts to address such external factors may be less likely to substantially decrease CVD risk in women with PCOS if that risk is driven primarily by biological factors such as hyperandrogenism.

In the general population, SDoH and social deprivation are also critical in the association between Black race and poor health outcomes, including mortality related to CVD. This highlights race as a social construct, with no racially based genetic etiology to observed disparities [13]. For example, a 2023 study of 252 218 US adults found that the higher CVD mortality in Black compared to White patients was no longer observed after adjusting for SDoH [14]. Furthermore, 40% to 60% of the association between race and mortality was mediated by SDoH burden. This emphasizes the critical contribution of SDoH to racial disparities in CVD and underscores the importance of population-level interventions to address adverse SDoH.

Prior work by the authors has demonstrated a racial disparity in MetSyn among women with PCOS, with a higher risk in Black compared to White women [4, 15]. Though the role of SDoH in the racial disparity in CVD is clear in the general population, it is unknown whether some of the higher risk of CVD and MetSyn in Black compared to White women with PCOS may be attributable to SDoH. Of note, the 2021 study by Berni et al described earlier did not report on the racial/ethnic characteristics of the cohort or include race/ethnicity as a potential confounder in the analyses. Understanding the role of SDoH in CVD risk in women with PCOS will guide whether interventions targeting 1 or more of these factors need to be part of their overall care delivery to reduce observed racial disparities.

Therefore, this study aimed to assess whether SDoH, as measured by the Social Deprivation Index (SDI), is associated with new-onset MetSyn in women with PCOS, and if so, whether this association contributes to the racial disparity in MetSyn between Black and White women with PCOS. The SDI is a neighborhood-level composite of several social determinants for a given geographical area that is commonly used as a proxy for SDoH, with higher SDI indicating greater social deprivation and more disadvantaged SDoH. While it does not indicate social determinants on an individual level, such as the education level of a given patient, it reflects the broader state of social and economic disadvantage of a patient's immediate environment and has been linked with a myriad of poor health outcomes. Utilizing a unique subset of well-characterized patients with access to care and multiple years of follow-up, this work provides crucial data on the role of SDoH in CVD risk in women with PCOS and the extent to which these factors mediate disparities in this population.

## Materials and methods

### Study population

We conducted a longitudinal cohort study of adult women seen at the Penn PCOS Center with at least 2 visits 3 years apart between 2008 and 2022. Diagnosis of PCOS was confirmed using the Rotterdam criteria and exclusion of mimicking conditions such as hypothyroidism and congenital adrenal hyperplasia [1]. As the interest was in new-onset MetSyn, patients were excluded if they met criteria for MetSyn at the baseline visit or did not have cardiometabolic screening at least 3 years apart. We included only patients with hyperandrogenic phenotypes, as these are most strongly associated with metabolic abnormalities [16].

### SDI

The SDI was determined for each participant using their documented zip code. The SDI ranges from 1 to 100, with higher scores indicating greater social deprivation [17]. The score is a composite of 7 characteristics identified using factor analysis, in which many candidate SDoH are consolidated into a smaller number of the most relevant variables. Data extracted from the 2016-2020 American Community Survey used to calculate the most recently available SDI included

- Percent living in poverty
- Percent with fewer than 12 years of education
- Percent single-parent households
- Percent living in rented housing units
- Percent living in overcrowded housing units
- Percent of households without a car
- Percent nonemployment among adults under 65 years

**Figure 1:**
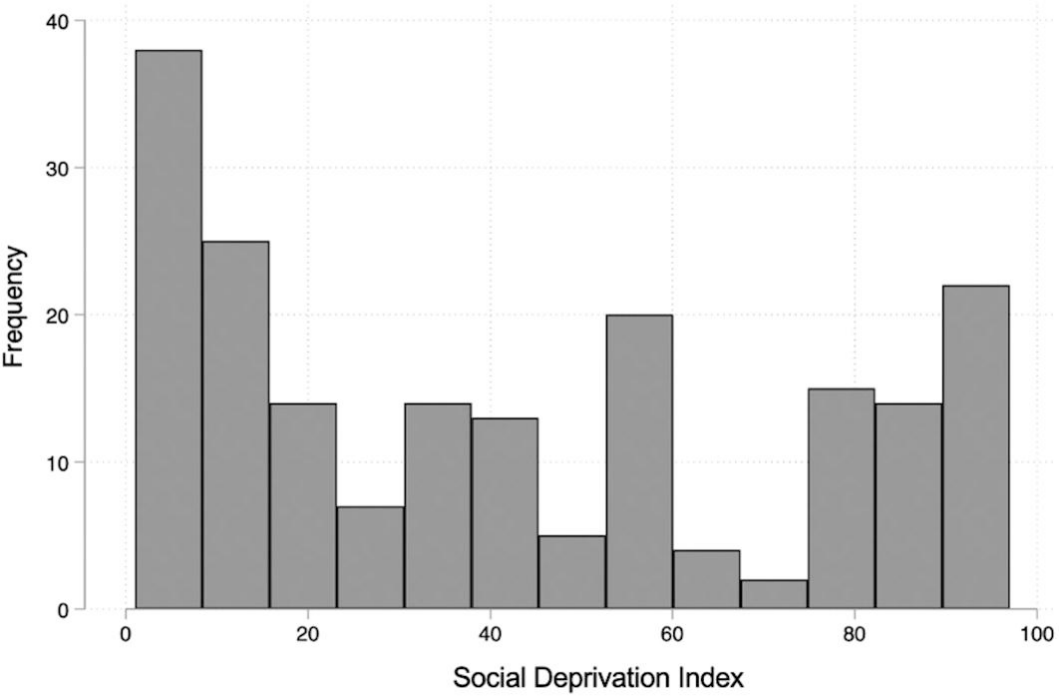
Distribution of the Social Deprivation Index in the study population.

The SDI was evaluated categorically, dividing the participants into high SDI (SDI ≥70) and low SDI (SDI <70) groups. This cutoff was chosen due to its use in prior studies of the SDI [18, 19]. Additionally, in our study population, the highest quartile included SDI scores of 72 or greater (Fig. 1), which is largely consistent with the cutoff used in the previous literature.

### Outcomes

The primary outcome was the risk of new-onset MetSyn during the study period. Participants who had a negative evaluation for MetSyn at their baseline visit but subsequently met criteria at any point during follow-up were considered to have developed new-onset MetSyn. MetSyn was defined as the presence of 3 or more of the following: BMI ≥30 kg/m^2^ as a proxy for increased waist circumference, hypertension (systolic blood pressure ≥130 mmHg or diastolic blood pressure ≥85 mmHg or taking medication for hypertension), dyslipidemia (high-density lipoprotein cholesterol <50 mg/dL or taking statin), hypertriglyceridemia (triglyceride levels ≥150 mg/dL or taking triglyceride-lowering medications), and hyperglycemia (fasting blood glucose >100 mg/dL or taking medication for diabetes) [20]. BMI ≥30 kg/m^2^ was used as a proxy for increased waist circumference as it is routinely measured in clinical care. This was based on prior studies using a similar methodology and the International Diabetes Federation's statement that if BMI is over 30 kg/m^2^, central obesity can be assumed and it is not necessary to measure waist circumference [4, 21, 22].

### Covariates

Data on covariates including demographic and clinical characteristics, biochemical features of PCOS, and medication prescriptions [combined oral contraceptive (COCP), metformin, and spironolactone] at baseline were collected via chart review.

### Race

Race was self-reported in the electronic medical record system. For the analysis of the association between SDI and incident MetSyn, all races were included. For the mediation analysis described later, only participants identifying as Black or White were included due to the small number of Asian and other participants as well as the less well-defined disparity in those racial groups.

### Statistical analysis

Descriptive statistics were performed to compare demographic and clinical characteristics between Black and White patients using 2-tailed *t*-tests or rank sum tests for continuous variables and chi-squared tests for categorical variables.

**Figure 2:**
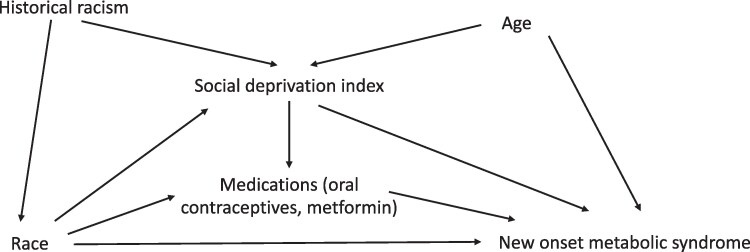
Directed acyclic graph depicting the association between race and metabolic syndrome.

A directed acyclic graph (DAG) was used to illustrate the relationship between SDI, new-onset MetSyn, and race (Fig. 2). Poisson regression with robust standard errors (SEs) was used to estimate the relative risk of new-onset MetSyn in participants with high vs low SDI adjusting for age, in line with the factors identified in the DAG. The primary analysis did not include BMI as a confounder because it was used as 1 of the criteria for MetSyn and is therefore part of the outcome. To examine for a BMI-independent effect of SDI on MetSyn, a sensitivity analysis was performed, including BMI (maximum documented during follow-up) in the multivariable model.

All analyses were performed in STATA 17 (StataCorp, College Station, TX). The *paramed* command was used to quantify the role that social deprivation plays in the association between race and new-onset MetSyn. This generated 2 models: first, a Poisson regression with robust SEs to estimate the risk of developing new-onset MetSyn by race and second, a logistic regression to estimate the association between high SDI and Black race. The *testparm* command was used to evaluate for an interaction between Black race and high SDI, and no significant interaction was identified (*P* = .57). Bootstrapping with 1000 replications was used to generate bias-corrected estimates. The proportion of the total error (TE) statistically accounted for by SDI was calculated as [log(RR_TE_)—log(RR_CDE_)]/log(RR_TE_). Age was identified as a potential confounder and therefore included in the multivariable model. Other covariates in the DAG were considered likely interrelated mediators between race and MetSyn or between SDI and MetSyn rather than true confounders of these associations. As such, they were not included in the primary analysis. However, an exploratory mediation model including prescription of COCPs, metformin, and spironolactone (in addition to age, as described earlier) was performed for comparison given their inclusion in prior studies [4]. It is important to note that SDI is a complex construct, and treating SDI as a single mediator is an oversimplification of this relationship; as such, these results should be interpreted descriptively rather than causally.

A 2-tailed *P*-value of <.05 was considered statistically significant.

## Results

A total of 222 participants met inclusion criteria and did not have MetSyn at baseline. Mean age at the baseline visit was 26.8 ± 6.3 years. Participants had a median of 6 visits (interquartile range 4-8) over 7 years (interquartile range 4-9). During follow-up, 97 (43.7%) participants developed incident MetSyn.

### SDI and new-onset MetSyn

The distribution of SDI scores is shown in Fig. 1. Baseline demographic and clinical characteristics are described for participants with high vs low SDI (Table 1). Overall, 59 (26.6%) participants had an SDI ≥ 70 (considered high SDI, indicating greater social deprivation). A larger proportion of participants with high SDI identified as Black (50.9%) compared to those with low SDI (11.7%) (*P* < .001). Other notable differences included higher BMI (*P* < .001) and free testosterone (*P* = .02) and a lower rate of prescription of COCPs (*P* = .04) in those with high SDI.

**Table 1:** Baseline demographic and clinical characteristics by SDI

	SDI <70 n = 163	SDI ≥70 n = 59
Age (years)	26.8 ± 5.1	26.8 ± 6.7
Years of follow-up	7.3 ± 2.8	6.6 ± 3.1
Race		
Asian	7 (4.3)	2 (3.4)
Black	19 (11.7)	30 (50.9)
Other	16 (9.8)	4 (6.8)
White	121 (74.2)	23 (39.0)
Hispanic/Latina	10 (6.1)	0
Insurance		
Private	155 (95.1)	9 (15.3)
Public	7 (4.3)	50 (84.8)
Body mass index (kg/m^2^)	28.3 ± 7.1	32.0 ± 7.0
Ever smoker	17 (10.4)	5 (8.5)
Weight (kg)	75.7 ± 19.6	85.1 ± 20.2
PCOS phenotype		
H + O + P	93 (57.1)	43 (72.9)
H + P	15 (9.2)	5 (8.5)
H + O	55 (33.7)	11 (18.6)
Total testosterone (ng/dL)	53.1 ± 22.6	61.9 ± 30.3
Free testosterone (pg/mL)	5.3 ± 4.0	7.6 ± 6.4
Combined Oral Contraceptives	72 (44.2)	17 (28.8)
Metformin	31 (19.0)	11 (18.6)
Spironolactone	30 (18.4)	6 (10.2)
Hemoglobin A1c (%)	5.3 ± 0.5	5.3 ± 0.5
Systolic blood pressure (mmHg)	119.4 ± 13.4	122.5 ± 14.2
Diastolic blood pressure (mmHg)	70.9 ± 7.5	72.2 ± 8.3
Fasting glucose (mg/dL)	84.9 ± 15.9	84.7 ± 8.4
High-density lipoprotein cholesterol (mg/dL)	59.7 ± 13.7	59.0 ± 15.3
Triglyceride (mg/dL)	90.0 ± 41.8	90.7 ± 41.1

Data are mean ± SD or number (percentage).

Abbreviations: H, hyperandrogenism; O, oligo-ovulation; P, polycystic ovaries on ultrasound; PCOS, polycystic ovary syndrome; SDI, Social Deprivation Index.

Participants with high SDI were more likely to develop MetSyn than those with low SDI [54.2% vs 40.0%, relative risk 1.36, 95% confidence interval (CI) 1.01-1.84, *P* = .04], including after adjustment for age (adjusted relative risk 1.42, 95% CI 1.07-1.91, *P* = .02).

### Black race, social deprivation, and new-onset MetSyn

Given prior studies demonstrating a higher risk of MetSyn in Black compared to White women with PCOS and the current findings demonstrating an association between high SDI and the development of MetSyn, a mediation analysis was performed to assess whether high SDI may play a role in the racial disparity. This analysis included 49 Black and 144 White participants. Of those, 6.1% and 4.9% were of Hispanic ethnicity, respectively (*P* = .73).

Consistent with previous evidence, Black participants were more likely to develop new-onset MetSyn than White participants (59.2% vs 37.5%, relative risk 1.58, 95% CI 1.15-2.16, *P* = .004). Table 2 presents the total and controlled direct effects of Black race on incident MetSyn, as well as indirect effects through high SDI. SDI accounted for 21.0% of the total effect of Black race on new-onset MetSyn when adjusting for age. Sensitivity analysis including BMI in the adjusted model resulted in a similar estimate (19.8%), and an exploratory model including age, COCP, metformin, and spironolactone as covariates also did not alter the proportion eliminated (20.2%).

**Table 2:** Direct and indirect effects between Black race and new-onset MetSyn

	Unadjusted relative risk new-onset MetSyn	Relative risk new-onset MetSyn adjusted for age	Relative risk new-onset MetSyn adjusted for age and BMI
Total effect of Black race on new-onset MetSyn	1.59 (1.17-1.24)	1.59 (1.12-2.20)	1.31 (0.82-2.10)
Controlled direct effect of Black race on new-onset MetSyn	1.45 (0.97-2.11)	1.44 (0.97-2.13)	1.24 (0.87-1.79)
Indirect association through SDI (%)	20.2	21.0	19.8

Abbreviations: BMI, body mass index; MetSyn, metabolic syndrome; SDI, Social Deprivation Index.

## Discussion

In this cohort of patients with hyperandrogenic PCOS and longitudinal metabolic testing within a tertiary healthcare system, we found that high SDI was associated with increased risk of developing new-onset MetSyn. Furthermore, high SDI may play a role in the association between Black race and incident MetSyn.

Though prior work had demonstrated the racial disparity in MetSyn among Black and White women with PCOS and studies in the general population have indicated a key contribution of SDoH to CVD, this is the first study to demonstrate that social deprivation may play a role in the observed disparities specifically in this high-risk population. These findings highlight the importance of assessing for social deprivation at the time of PCOS diagnosis, when initial metabolic screening is recommended. Identification of barriers to care that underlie the higher risk of developing new-onset MetSyn may allow for the provision of appropriate support and increased screening, particularly in Black compared to White patients.

The population of patients included in this study may not be representative of the full spectrum of social deprivation, as the longitudinal study design necessitated ongoing engagement with the healthcare system and adherence to recommended screening guidelines. Patients whose high social deprivation precluded them from presenting for care were likely not included, as they were not able to be evaluated for the outcome of interest. Future studies are needed to identify the patients who, perhaps because of high social deprivation, do not receive longitudinal (or any) care, as this is likely a group of particularly high-risk individuals. Engagement of these patients should be a priority of future studies.

While the mediation models suggest that SDI accounts for a substantial proportion of the association between Black race and new-onset MetSyn, these results should be interpreted as associative rather than causal given the multifactorial nature of racial disparity. As depicted in the DAG (Fig. 2), there are likely numerous other mediators between Black race and MetSyn as well as high SDI and MetSyn that merit further examination. Many of these mediators are likely also interrelated and stem from a common source of structural racism. Furthermore, SDI is an area-level metric and does not fully capture individual-level deprivation or experiences of discrimination, which have been independently associated with worse cardiometabolic outcomes in the general population [23]. Future studies should explore more granular measures of social deprivation, including individual socioeconomic position, perceived racism, and healthcare access.

The 2023 international evidence-based guidelines for PCOS recommend universal metabolic screening with lipid profile, glycemic status, weight, and blood pressure at the time of diagnosis, with subsequent periodic assessment [1]. The importance of these metabolic parameters is well recognized in this high-risk population, and they have been the primary target of interventions. For example, lifestyle management is the first-line approach to weight management to reduce the risk of obesity. However, there are limited data on other factors that may be upstream of these metabolic abnormalities, including the neighborhood-level SDoH captured by the SDI. While the SDI does not pinpoint specific factors or policies to target, as it is a composite score, the results of this study highlight the need to look beyond the traditional CVD risk factors to reduce the risk of MetSyn as well as the racial disparity in MetSyn in women with PCOS. For example, our findings support the importance of public health policies that target neighborhood-level social deprivation, such as increasing employment, affordability of living space, and education, to reduce the observed racial disparity in MetSyn [24, 25].

Strengths of this study include the novel examination of social deprivation in patients with PCOS, longitudinal study design, and well-defined cohort of patients with hyperandrogenic PCOS. Limitations of this study include the exclusion of races other than White and Black in the mediation analysis. This was primarily driven by the smaller number of participants identifying as other races, as well as the less well-established racial disparity in MetSyn among these groups. Though Hispanic women with PCOS have been shown to have a higher risk of MetSyn than non-Hispanic women, SDI did not differ by ethnicity in our study and therefore ethnicity was not included as an additional potential mediator [26]. Additionally, the potential inaccuracy of race data in the electronic medical record is a weakness, and ongoing efforts to decrease misclassification are needed [27].

An additional limitation is that, as described here, SDI is only 1 method by which to examine SDoH and is not comprehensive. Though residence in neighborhoods with high social deprivation does correlate with more disadvantaged individual SDoH (health literacy, health insurance status, education, etc.), the SDI ultimately reflects neighborhood-level, structural SDoH and not individual SDoH [28]. However, prior studies have demonstrated the importance of neighborhood-level SDoH, and using an easily measured and accessible metric such as the SDI is an important first step that provides impetus for further, more granular investigation [29]. Lastly, we did not have the data to assess downstream biochemical effects of social deprivation that could be on the pathway to MetSyn, such as C-reactive protein as a marker of chronic inflammation [30].

## Conclusions

In this longitudinal study of patients with hyperandrogenic PCOS, we identified an association between high social deprivation and increased risk of developing MetSyn. Furthermore, we found that social deprivation may play a role in the racial disparity in MetSyn between Black and White women with PCOS. While more studies are needed to fully elucidate the importance of individual SDoH, the results of the present study highlight the important role of neighborhood-level factors in the development of new-onset MetSyn within a high-risk population.

## Data Availability

Some or all datasets generated during and/or analyzed during the current study are not publicly available but are available from the corresponding author on reasonable request.
